# Nurses’ experiences of self-management support for adults with tuberculosis and human immunodeficiency virus coinfection

**DOI:** 10.4102/hsag.v29i0.2546

**Published:** 2024-04-19

**Authors:** Eric Tornu, Portia J. Jordan, Michael McCaul

**Affiliations:** 1 Department of Nursing and Midwifery, Faculty of Medicine and Health Sciences, Stellenbosch University, Cape Town, South Africa; 2 Department of Global Health, Centre for Evidence-based Health Care, Division of Epidemiology and Biostatistics, Stellenbosch University, Cape Town, South Africa

**Keywords:** adult, barriers, facilitators, HIV, nurse, self-management, support, tuberculosis

## Abstract

**Background:**

Professional nurses provide self-management support to adults (18 years and older) living with tuberculosis (TB) and human immunodeficiency virus (HIV) coinfection to enable them to mitigate its impact on their lives. However, the experiences of professional nurses providing self-management support to adults with TB-HIV coinfection remain unclear.

**Aim:**

This study explored and described the experiences of professional nurses on the provision of self-management support to adults living with TB-HIV coinfection in Greater Accra, Ghana.

**Setting:**

Three public primary health facilities in Greater Accra, Ghana.

**Methods:**

An exploratory, descriptive qualitative design was used. Twenty-two purposively sampled professional nurses were interviewed face-to-face individually using an interview guide. Interviews were recorded with participants’ permission, transcribed and analysed thematically using MAXQDA software.

**Results:**

The three themes generated revealed that the: (1) self-management problems of adults living with TB-HIV coinfection included their recurring physical, mental and social problems, (2) the support provided to adults with TB-HIV coinfection included symptom, nutritional, medication and psychosocial self-management support, (3) the factors related to providing self-management support showed that self-management support was influenced by patient, nurse and health facility-related factors but was feasible, equitable and acceptable to patients and stakeholders.

**Conclusion:**

Professional nurses’ self-management support practice entailed improvising limited resources to address the recurring problems of adults living with TB-HIV coinfection. Nurses require adequate resources to provide comprehensive self-management support.

**Contribution:**

The contextual evidence provides insight into the self-management problems of adults with TB-HIV coinfection and the factors influencing professional nurses’ self-management support.

## Introduction

Tuberculosis (TB) is the leading cause of death among persons living with human immunodeficiency virus (HIV) infection globally (UNAIDS 2022). In 2021, 187 000 people living with tuberculosis-human immunodeficiency virus (TB-HIV) coinfection died worldwide, 136 000 died in Africa and 37 000 deaths were recorded in Ghana (World Health Organization 2022). For adults (18 years and older) living with TB-HIV coinfection, avoiding complications and death requires that they mobilise all the support they can get from relatives and professional nurses to self-manage the impact of TB-HIV illness and treatment on their lives (Sullivan & Nathavitharana 2022).

Self-management refers to the day-to-day tasks that persons living with chronic conditions carry out to deal with its effect on their lives. Self-management support entails the support provided to individuals to self-manage their chronic conditions (Davis et al. 2022). Professional nurses are formally trained and licenced to provide nursing care (International Council of Nurses 2020). Professional nurses’ nursing care for patients includes assessing their health problems, educating them about their illnesses and treatment and administering medications (Ghana Health Service 2014).

The existing studies suggest that professional nurses provide self-management support to adults living with TB and HIV by equipping them with the requisite knowledge, skills and confidence to self-manage the effect of TB and HIV coinfection on their well-being (Areri, Marshall & Harvey 2020; Baluku et al. 2023; Selasa, Israfil & Teli 2021). Furthermore, professional nurses noticed that their self-management support was promoted by their training and self-confidence but inhibited by insufficient time and support from team members (Duprez et al. 2018).

However, there is a dearth of studies that have examined the experiences of professional nurses regarding the self-management support they provide adults with TB-HIV coinfection in TB-HIV clinics, including the factors that promote or inhibit their self-management support. The findings can provide insight into professional nurses’ TB-HIV coinfection self-management support to inform TB-HIV coinfection-related care, research and policy formulation. This study aimed to explore and describe the experiences of professional nurses on the provision of self-management support to adults living with TB-HIV coinfection in Greater Accra, Ghana.

## Research methods and design

### Study design

The qualitative, exploratory and descriptive research study explored and described the experiences of professional nurses regarding the self-management support they provide to adults with TB-HIV coinfection in TB-HIV clinics (Hunter, McCallum & Howes 2018).

### Study setting

The study was conducted in three purposively sampled health facilities in the Greater Accra region of Ghana to gain access to professional nurses who provide care to adults with TB-HIV coinfection. The three public health facilities included one teaching hospital and two polyclinics (A and B), which offer primary healthcare services and integrated TB-HIV treatment to adults with TB-HIV coinfection from Mondays to Fridays. The Greater Accra Region is Ghana’s predominantly urban capital, situated near its southern border (Ghana Statistical Services 2019). All three health facilities (study sites) operate TB-HIV clinics on an outpatient basis with professional nurses who are stationed in consulting rooms. The professional nurses work with physicians, laboratory technicians and pharmacists and provide integrated TB-HIV treatment care to adults with TB-HIV coinfection by conducting voluntary counselling tests for TB and HIV diagnosis and administering antituberculosis and antiretroviral medication (Anku et al. 2020).

### Population and sampling

Twenty-two professional nurses who provide care to adults with TB-HIV coinfection in the TB-HIV clinics of three public health facilities within Greater Accra, Ghana, were purposively sampled to provide their experiences on self-management support. The professional nurses were included in the study if they had cared for adults with TB-HIV coinfection (aged 18 years and older) in one of the three purposively sampled study sites for at least 1 month. Professional nurses who exclusively provide inpatient care in the wards were excluded from the study. The inclusion and exclusion criteria ensured that only credible participants (professional nurses) who had provided care to adults with TB-HIV coinfection in TB-HIV clinics within the study setting shared their experiences on TB-HIV coinfection self-management support.

A total of 22 professional nurses participated in the study. The sample size was informed by information power, which suggests that ‘the more relevant information a sample holds, the fewer participants are needed’ (Braun & Clarke 2019:210). The participants had previous experience and relevant information regarding self-management support. However, the researcher obtained redundant data (repetitive information) after interviewing the 20th participant, which indicated that data saturation had been attained. Two more interviews were conducted, which confirmed that no new information would be obtained after additional interviews.

Participants were recruited from each study site upon institutional approval and with the assistance of the health facilities’ TB-HIV coordinators. The first author (E.T.) approached each potential participant individually (face to face) and explained the study’s purpose and procedures to them. The researcher obtained each participant’s written consent and agreed on the interview dates and venues.

### Data collection

Data were collected from December 2021 to December 2022 with an interview guide developed by the authors (E.T., P.J. and M.M.) and informed by the study objectives, literature and the 5A model of self-management support (Glasgow et al. 2003). The male researcher (E.T.) and a trained female research assistant (AL), who was a graduate professional nurse, conducted the face-to-face individual semi-structured individual interviews separately. The researcher interviewed 20 participants and the research assistant interviewed the remaining two. The researcher trained the research assistant on the study’s procedures, data collection and ethical research conduct.

Data were collected by interviewing each participant once in a room within the participants’ health facility, at their preferred time, and in their preferred language (English). The interviews lasted an average of 46 minutes and were audio-recorded with participants’ permission. Participants completed a demographic characteristic form after the interview. The researcher’s observations made during interviews were documented in field diaries and used to inform subsequent interview probes and data analysis (Creswell & Poth 2016). The interviewers’ observations included the participants’ non-verbal cues, such as pauses in speech, strength of vocal expression and body language, which may signify important aspects of their experiences (Denham & Onwuegbuzie 2013). The researcher also held one face-to-face debriefing meeting with the research assistant after her data collection to obtain her interpretation of the data and inform the researcher’s data analysis. The debriefing meeting covered the participants’ recurring responses during the interviews.

### Data analysis

The audio-recorded interviews were transcribed verbatim for concurrent data collection and analysis with MAXQDA data analysis software. The qualitative data were analysed using reflexive thematic analysis through data familiarisation, coding, theme generation, refinement and reporting (Braun & Clarke 2020). The researcher read the transcripts several times to familiarise himself with the data. The researcher then grouped participants’ responses into meaningful codes. The related codes were grouped into sub-themes, and sub-themes were organised into themes. The themes were refined by rewording and regrouping before reporting them (Braun & Clarke 2020). An independent coder conducted separate data analyses to limit confirmation bias in the analysis. The researcher, an independent coder and supervisors (P.J. and M.M.) discussed the analysed data till a consensus was attained.

### Ethical considerations

The study was conducted in line with the Helsinki Declaration of 1964 and its subsequent revisions. Ethical approval was obtained from the ethics committees of a public university in South Africa (S21/05/094), a public hospital in Ghana (000144/2021) and a health institution in Ghana (004/09/21). The researcher explained the purpose and procedures of the study to participants and answered their questions before obtaining their written informed consent to participate in the study, record interviews and publish anonymised findings. Participants’ involvement was voluntary, and they could withdraw from the study at any time without any penalty. Participants’ confidentiality and privacy were ensured by maintaining anonymity and not eliciting their names during interviews or on demographic characteristic forms. The risk of harm was kept to the minimum by building rapport with the participants, asking non-sensitive questions and arranging for a clinical psychologist to intervene if any participant experienced an emotional breakdown. No emotional breakdown occurred among participants throughout the study. All soft copy data (such as interview audio recordings) were kept on a password-protected pen drive and locked together with hardcopy documents in a cabinet in the researcher’s office. Data will be kept for 5 years for verification and deleted or shredded before permanent disposal (Flick 2022).

### Trustworthiness

Trustworthiness was ensured using the principles of credibility, transferability, dependability and confirmability (Cypress 2017; Lincoln & Guba 1985). Credibility was ensured by using accepted qualitative research techniques and through prolonged participant engagement by interviewing 22 professional nurses who shared their self-management support experiences freely. The study was also undertaken under a qualitative research specialist’s guidance (P.J.). Two individual participant interviews were conducted to pretest and enhance the data collection tool (interview guide). Member checking was conducted by repeating participants’ comments for their confirmation or clarification, thus ensuring that the information obtained during interviews was accurate (Creswell & Poth 2016). The researcher’s data analysis was compared with an independent coder’s analysis to ensure that conclusion drawn from the data were reliable. The researcher used a reflexive field diary throughout the study to control any preconceptions about the participants or self-management support. Dependability was ensured through a thick description of the data collection and analysis procedures. Interview recordings were checked with the participants’ quotes to ensure consistency in meaning. Confirmability was ensured by comparing the researchers’ findings with the independent coder’s findings and discussing them with the study supervisors (P.J. and M.M.) till consensus was attained. An audit trail was maintained to document and confirm how data were collected and analysed. The participants’ verbatim quotes were reported with findings to confirm the conclusions made. Transferability was ensured by explicitly documenting and describing the study background, participants, context, data collection, and analysis (Grove & Gray 2018).

## Results

### Participants’ characteristics

The researcher contacted 22 professional nurses who agreed to participate in the study (represented by PN 1 to PN 22). Eight participants worked in the TB-HIV clinics of the teaching hospital; nine were from Polyclinic A, and five were from Polyclinic B. The participants’ ages ranged from 26 to 54 years, and 20 were female. The participants’ characteristics are summarised in Table 1.

**TABLE 1:** Characteristics of professional nurses.

ID	Age (years)	Sex	Duration of managing AWTB-HIV
PN 1	30	Male	6 years
PN 2	32	Female	5 years
PN 3	42	Female	7 years
PN 4	54	Female	17 years
PN 5	34	Female	11 years
PN 6	27	Male	3 years
PN 7	31	Female	3 years
PN 8	33	Female	3 months
PN 9	27	Female	6 months
PN 10	26	Female	1 year
PN 11	28	Female	1 year
PN 12	32	Female	5 years
PN 13	27	Female	3 years
PN 14	36	Female	7 years
PN 15	32	Female	10 years
PN 16	50	Female	6 years
PN 17	39	Female	1 year
PN 18	39	Female	1 year
PN 19	40	Female	4 years
PN 20	34	Female	7 years
PN 21	35	Female	3 years
PN 22	27	Female	1 year

AWTB-HIV, adults (18 years and older) living with tuberculosis-human immunodeficiency virus.

### Themes and subthemes

The three main themes and 10 sub-themes found are summarised in Table 2.

**TABLE 2:** Table of themes, subthemes and codes.

Themes		Sub-themes
1.	Self-management problems of adults living with TB-HIV coinfection	1.1 Recurring physical health problems of TB-HIV coinfection 1.2 Mental health problems of living with TB-HIV coinfection 1.3 Social interaction problems with others
2.	Support provided to adults with TB-HIV coinfection	2.1 Symptom self-management support 2.2 Medication and nutrition support 2.3 Psychosocial self-management support
3.	Factors related to providing self-management support	3.1 Feasibility of self-management support 3.2 Equity of self-management support 3.3 Acceptability of self-management support 3.4 Facilitators and barriers of self-management support

TB-HIV, tuberculosis-human immunodeficiency virus.

#### Theme 1: Self-management problems of adults living with tuberculosis-HIV coinfection

This theme describes participants’ experiences regarding the self-management problems of adults with TB-HIV coinfection. The three sub-themes generated from this theme were: (1) recurring physical health problems of TB-HIV coinfection, (2) mental health problems of living with TB-HIV coinfection and (3) social interaction problems with others.

**Recurring physical health problems of tuberculosis-HIV coinfection:** Participants mentioned that adults with TB-HIV coinfection experienced physical health problems related to resolving TB and HIV symptoms, managing multiple medications, overcoming nutritional difficulties and practising infection prevention. According to participants, the recurring TB-HIV symptoms included persistent cough, weight loss, body pains, weakness, diarrhoea and pallor. The adults with TB-HIV coinfection also had difficulties obtaining their multiple medications, managing unpleasant side effects (such as itching skin, urine discolouration and vomiting) and adhering to the medication when they felt better:

‘The person was coughing … having herpes, watery stools, vomiting, sunken eyes because of the passing out of the watery stool, the person was pale as well, and halitosis … Taking these huge drugs and combining them is a problem … I am talking about TB drugs and antiretroviral drugs … The drugs for the intensive phase are big in size.’ (PN 1, 30 years, Male)

‘When they realise they are doing okay, they are doing well, they tend to break … They start well, but they definitely break up.’ (PN 2, 32 years, Female)

Participants added that adults with TB-HIV coinfection were often malnourished, lacked knowledge on what to eat to gain weight and their medications made them hungrier. Furthermore, the immunocompromised patients were at high risk of opportunistic or sexually transmitted infections, yet struggled to adopt infection prevention practices. Their self-management problems necessitated professional nurses’ support to overcome them:

‘Most of them physically they are malnourished and the medications also go with food. You can’t take medicine and then without eating well … It makes them also eat.’ (PN 12, 32 years, Female)

‘The very first month I met the guy [*AWTB-HI V*], the wife was having STI [*sexually transmitted infections*] … they were re-infecting themselves, so we have to treat the woman and treat the husband.’ (PN 4, 54 years, Female)

**Mental health problems of living with tuberculosis-HIV coinfection:** Participants indicated that adults with TB-HIV coinfection had mental health problems such as difficulties in: (1) accepting TB-HIV diagnosis, (2) controlling their emotions and suicidal thoughts, (3) obtaining accurate knowledge, (4) overcoming substance abuse and (5) gaining confidence to self-manage TB-HIV coinfection. They noticed that the patients were often shocked by their TB-HIV diagnoses. They had difficulty accepting a lifelong diagnosis of HIV and experienced depressed mood, fear, anxiety, loss of interest and suicidal thoughts:

‘Somebody is taken unawares … had to go through– three months before she accepted to take medication for herself … Most of them refuse to accept that this is what I am going to live with the rest of my life. It’s difficult to accept the fact that they have HIV.’ (PN 2, 32 years, Female)

‘Like for an hour she [*adult with TB-HIV coinfection*] couldn’t stop crying, she went home, she even brought whatever she was going to take, she brought it here. she was going to take it in front of us … a poisonous substance.’ (PN 20, 34 years, Female)

Participants revealed that some adults with TB-HIV coinfection abused alcohol and cannabis. They also lacked accurate knowledge about TB-HIV coinfection, its cause, treatment and self-management, even after receiving health education, necessitating support from professional nurses:

‘When you give them money to buy food and eat in order to take their drugs, they use it on alcohol and smoking.’ (PN 1, 30 years, Male)

‘When they come, you ask of all the information that the doctor had told them … you are assessing patient knowledge about even the medication, his own condition, it’s zero.’ (PN 5, 34 years, Female)

**Social interaction problems with others:** The adults with TB-HIV coinfection had problems disclosing their health status to their relations, mobilising social support and adjusting to life with multiple infectious chronic conditions. The participants explained that apart from female older patients (aged 50–60 years), most adults with TB-HIV coinfection hesitated in disclosing their TB-HIV status to their partners (spouses) or relations because they did not want to be abandoned, exposed or stigmatised. The patients’ status non disclosure limited their access to emotional and financial social support from their relations. The adults with TB-HIV coinfection also had to moderate their social activities and adopt lifestyle changes (such as limiting sexual partners and avoiding crowded settings), which they had difficulty continuing without professional nurses’ support:

‘The youth, they are hiding it [*TB-HIV diagnosis*] and those that they’re married and their partner don’t know, most of them they are hiding it from them but the adults, for our grandmothers …let me say from 60 [*years*] or 50 [*years*] going, most of them their children used to bring them here, so one or two know that their mother or their father is having this disease.’ (PN 11, 28 years, Female)

‘They feel ‘you are bringing a new thing into my life’, so the moment clients start getting better, if you don’t take care, clients will avoid the new thing you are introducing … The social life of the client, you must deal with it.’ (PN 4, 54 years, Female)

#### Theme 2: Support provided to adults with tuberculosis-HIV coinfection

This theme describes the participants’ experiences regarding the self-management support activities they carried out for adults with TB-HIV coinfection to overcome their self-management problems. The three sub-themes identified were: (1) symptom self-management support, (2) medication and nutrition support and (3) psychosocial self-management support.

**Symptom self-management support:** The participants supported the adults with TB-HIV coinfection to self-manage their symptoms by asking about them and assessing (examining) them during clinic visits. Participants advised the patients and relations on how to manage symptoms by resting to reduce stress, drinking oral rehydrated salts to manage diarrhoea and eating a balanced diet to correct anaemia or prevent recurrent infections. The professional nurses also provided analgesics (for body pains), antihistamines (for rashes and itch) and appetite stimulants to improve the patients’ appetite. Where necessary, the adults with TB-HIV coinfection with recurrent or severe physical symptoms were referred to other healthcare providers (clinicians, dermatologists and psychologists) for further management or hospitalisation:

‘Sometimes the person says she has reduced weight, and she is having back pain … so when it is headache you take Para[*cetamol*], when is diarrhoea you take ORS [*oral rehydrated salts*] and Zinc tablets … but if it is severe, you turn to the clinic so that you go and see the doctor.’ (PN 10, 26 years, Female)

‘So sometimes we encourage them not to do too much than they can, like you tell them that if at first, … you walk a little distance, you have to sit down to reduce your labour, the amount of work you do, you should try and reduce it.’ (PN 15, 32 years, Female)

**Medication and nutrition support:** The participants supported the adults with TB-HIV coinfection to start and manage their TB-HIV treatment by assessing what they knew about their treatment, allowing them to ask questions and providing health education to reinforce their knowledge and medication adherence. They also advised the adults with TB-HIV coinfection about when and how to take antituberculosis and antiretroviral medication to ensure maximum effectiveness and reduce medication misuse. The participants also trained the patients to identify and manage medication side effects should they occur. They supported the adults with TB-HIV coinfection to overcome adverse medication side effects by administering counteracting medication, changing their medication combination or referring them to physicians for further treatment:

‘Sometimes we would even ask them “what’s the time for taking the medication” and you see that they are sitting there …” what are some of the things you’d feel when you take the medicine?” At least you should mention one so if there are no responses then you know they don’t have enough knowledge and we mostly do health talks.’ (PN 12, 32 years, Female)

‘For the TB medication, there are signs, or let’s say, side effects of the drug. That one, we teach you that when you take it, maybe you get a discolouration of the urine, you can get itchiness and we’ll give you something to counteract it. So that when you are getting those effects, you know that it’s because of the drug I am taking.’ (PN 2, 32 years, Female)

The participants served patients’ TB and HIV medications during clinic visits and delivered the medications to patients’ homes if they could not come for medication refills. Participants also followed up on the adults with TB-HIV coinfection to ensure medication adherence by calling them on the phone, conducting follow-up home visits, or sending treatment supporters (relations, volunteers and community nurses) to assist them in taking their medication. They also provided the patients with food or transportation money when a lack of funds prevented them from taking their medication as prescribed:

‘[*We*] give them the drugs to take them home and they come after taking the medication … we sometimes give them a week or we sometimes give them two weeks.’ (PN 6, 27 years, Male)

‘We save their numbers and call them. Sometimes too we send treatment supporters there to go and support … it involves people who have taken the medication in the past and survivors and nurses. So it can be general nurses or public nurse or the community health nurses.’ (PN 15, 32 years, Female)

‘those who are willing to take it complain about not having enough time to come for their medication. sometimes we transport it to them … some of them we meet them at their work place or at their homes. Some too are not around in the region, we transport via transport means like sending a parcel to another place.’ (PN 7, 31 years, Female)

‘I quite remember a client … he used to ask for money to buy porridge before he can take the drug early in the morning, so if we too we have it [*money*], we give it out.’ (PN 11, 28 years, Female)

The participants guided the adults with TB-HIV coinfection to manage their nutritional problems by assessing their weight and nutritional status during clinic visits. They counselled the patients to eat a balanced diet and emphasised the medications they should avoid. The professional nurses encouraged the intake of more fruits and vegetables, adequate water and less salty food. They referred some adults with TB-HIV coinfection to the nutritionist for additional counselling on nutrition or correction of malnutrition. They also provided food supplements for patients who could not afford food. Some food supplements included cereals such as *Tom brown* or *Weanimix*, fortified blended foods and plumpy nuts. The participants explained that although the food supplements were inadequate, they contributed immensely to the patients’ recovery:

‘We give them weanie mix, that is the Tom brown, for some cases if there are some we give but if there are none, we educate them to ensure the intake of fruits and vegetables … these are nutrients, they are highly dense nutrients that are made to help the patient, [*such*] as when they come [*and*] their body weight is not encouraging. So we give them this additional nutritious meal to help them to recover or enhance their nutritional status.’ (PN 6, 27 years, Male)

‘Sometimes we ask them if they are able to eat well … If they need to see a doctor, we refer them.’ (PN 9, 27 years, Female)

**Psychosocial self-management support:** The participants supported the adults with TB-HIV coinfection to overcome stigma and gain psychosocial support from their relations, religious groups and community members by: (1) encouraging status disclosure to supportive relations, (2) educating relations to support them, and (3) providing their phone numbers or contacts of volunteers to support them. The participants enhanced the patients’ confidence in their ability to self-manage by showing them their improved laboratory results or the number of folders of other patients receiving care at the TB-HIV clinic:

‘We save their numbers and call them. sometimes too we send treatment supporters there to go and support.’ (PN 15, 32 years, Female)

‘Through education, we let them gain that confidence … before we even put you on medication, we should show you the number of people [*we are*] taking care of; their folders. So it means people are living with HIV.’ (PN 7, 31 years, Female)

The participants encouraged the adults with TB-HIV coinfection to adhere to their religious beliefs by praying or attending church; however, they were not to take herbal concoctions or stop their prescribed medications:

‘We don’t encourage taking herbal medication and our medications at the same time … they can do their prayers and their medication they shouldn’t stop.’ (PN 17, 39 years, Female)

#### Theme 3: Factors related to providing self-management support

This theme describes participants’ experiences regarding their provision of self-management support to adults with TB-HIV coinfection and the factors that influenced it. The four subthemes identified were: (1) feasibility of self-management support, (2) equity of self-management support, (3) acceptability of self-management support and (4) facilitators and barriers of self-management support.

**Feasibility of self-management support:** Most participants observed that it was feasible for professional nurses to provide self-management support to adults with TB-HIV coinfection within the TB-HIV clinics in Greater Accra because self-management support was their duty, and they had been trained to support the patients; therefore, they could not leave them alone without supporting them. The participants noted that self-management support was a routine activity that professional nurses undertook in the TB-HIV clinics. They added that self-management was feasible because community health nurses assisted with follow-up home visits, and they improvised or tailored their self-management support to the needs of adults with TB-HIV coinfection:

‘It is feasible … because you cannot leave them alone. You need to help them. That is why you went through the training.’ (PN 3, 42 years, Female)

‘It is feasible to provide the support. It being feasible is that we can change the support each and every time, that is, in any other form or in any other way, we can switch whatever support that we can.’ (PN 6, 27 years, Male)

Although most participants indicated that self-management support was feasible, a participant explained that it was not feasible for professional nurses to provide self-management support to adults with TB-HIV coinfection in her facility because there were few professional nurses to provide support to the large population of patients who sought care at her TB-HIV clinic. She acknowledged that the number of patients decreased after 6 months of successful TB treatment; nonetheless, more staff were required in the TB-HIV clinic to provide the self-management support the adults with TB-HIV coinfection require:

‘No, [*be*]cause we are few at the TB HIV clinic … and the clients are amazing, amazingly high. We can’t even share them into fifties. We have lots of patients here … that’s that’s our problem we don’t have enough staff … the thing is with the adult with TB HIV, after six months you let them off, they’re cured … So they will be high but as time goes on the number will decrease.’ (PN 12, 32 years, Female)

**Equity of self-management support:** Most participants noticed that professional nurses could provide equitable self-management support to adults with TB-HIV coinfection within the TB-HIV clinics because: (1) they provide as much support as each patient and relations required, (2) TB-HIV treatment and nursing support were free for all adults with TB-HIV coinfection and (3) they scheduled the patients on different clinic days to manage the high numbers:

‘Nurses are, we are doing all the work, aahan. And we have, we’ve even started already. We do it all, everyday, we call patients whenever they bring their spouses or anything, we do the health education, we test them … we follow up on them about the side effect of drugs … so basically we are even doing it already, so its not a new thing.’ (PN 14, 36 years, Female)

‘they all don’t come in a day at least we have appointment days that is when you can have some time for a patient … definitely whatever support that we need we try to give whereby necessary.’ (PN 7, 31 years, Female)

**Acceptability of self-management support:** Most participants indicated that self-management support was acceptable to patients and stakeholders because the adults with TB-HIV coinfection want to get better and live longer. Other patients accepted self-management support readily because peer educators recommended professional nurses’ self-management support and the patients trusted the support professional nurses provided in primary health facilities:

‘we give the support to the patient, they do take it and because they want to get better they do take it …, it is very acceptable.’ (PN 6, 27 years, Male)

‘Men sexing [*who have sex with*] men, this FSW, female sex workers and their partners … the peer educator encourages them that ‘ooh if you come to this place the nurse who takes care of you is cool, you won’t have any issues’. That is when they open up for you to support them.’ (PN 7, 31 years, Female)

**Facilitators and barriers of self-management support:** Participants observed that the factors that facilitated self-management support were: (1) patients’ acceptance of TB-HIV diagnosis and relations’ social support, (2) professional nurses’ empathy, motivation, knowledge, skills, and training on self-management support, as well as (3) availability of funds and reduced waiting times at the TB-HIV clinic:

‘Factors like somebody, or a relation, or a friend accepting the patients with the condition. Volunteering that, no matter what, I am going to be by your side. It really helps us … Availability of maybe some foods like the Tom Brown we used to give. If we will still get it to support the patient, it will also help us.’ (PN 2, 32 years, Female)

‘If you’re empathetic and give the willingness, you have the willingness to support your client at any time.’ (PN 4, 54 years, Female)

‘motivation of the staff is key … if nurses are motivated with money at the end of the month to cater for transportation it will motivate us.’ (PN 1, 30 years, Male)

‘knowledge and skills … for the nurse without training and without having enough knowledge on what to do for a patient, you might not be able to give out what the patient needs.’ (PN 3, 42 years, Female)

Contrariwise, the patient-related factors that inhibited professional nurses’ self-management support were their high level of education, young age, non-compliance with advice and living long distances away from the health facility:

‘[*It*] is difficult managing the educated people too, because they will tell you they know their right.’ (PN 1, 30 years, Male)

‘the aged people, they are not stubborn like the teenage, the youth … because the aged they can see that now she is grown up so if he or she doesn’t take the drug he might die … Sometimes too distance … maybe the person is staying at Kaneshie now the person has moved to Kasoa, you see, so sometimes too distance and financial too.’ (PN 10, 26 years, Female)

In addition, patients’ unemployment was a barrier to professional nurses’ self-management support because they lacked income to fund self-management activities. Furthermore, the patients’ busy travelling schedules and poor communication with the professional nurse inhibited their self-management support:

‘dealing with somebody who doesn’t have anybody – any work to do, nor somebody to help them out, that one becomes a problem.’ (PN 2, 32 years, Female)

‘Some too, you call and there is no apparent reason why he’s not coming. Some will tell you, “Oh, I’ve travelled.” … Communication barrier is one because once patient doesn’t understand what he’s going in for, will not give you all the necessary cooperation.’ (PN 5, 34 years, Female)

The participants also observed that the health facility’s inadequacy of staff, space and logistics (such as nutritional supplements) were barriers to professional nurses’ self-management support to the adults with TB-HIV coinfection:

‘our unit here, ideally it is supposed to be spacious and big and airy as well but you don’t find it as such so this is a barrier to providing this support to the patient … the staff in here are not that many … you can’t get that adequate time to do that effective counselling … that is also a challenge to us.’ (PN 6, 27 years, Male)

## Discussion

From the findings, it was evident that adults with TB-HIV coinfection experienced self-management problems, which inhibited their self-management, requiring professional nurses’ self-management support. Previous studies among adults living with TB and HIV corroborate the findings that adults with TB-HIV coinfection experience physical health problems such as recurring symptoms, high medication burden, and nutritional difficulties (Areri et al. 2020; Selasa et al. 2021). As found in Uganda, the adults with TB-HIV coinfection also contended with unpleasant medication side effects such as urine discolouration and skin rash or itch, which inhibited their medication adherence (Nabisere-Arinaitwe et al. 2023). However, this study’s findings highlight the catastrophic impact of TB-HIV coinfection on the mental and social well-being of the adults with TB-HIV coinfection. Such that after accepting the long-term diagnosis and treatment of TB-HIV coinfection, adults with TB-HIV coinfection would have to permanently adjust to life with multiple chronic conditions by: (1) overcoming erroneous beliefs, (2) seeking accurate knowledge, and (3) mobilising social support through status disclosure (Hollingdrake et al. 2021). These life adjustments occurred while the adults with TB-HIV coinfection experienced stigmatisation from their relations or community members and could potentially impair their ability to self-manage (Areri et al. 2020; Nabisere-Arinaitwe et al. 2023).

As found in a study among Pakistanis living with multiple chronic conditions in the United Kingdom (UK), the patients’ health, family and beliefs were central to their self-management (Sultan & Swinglehurst 2021). This realisation may have informed professional nurses’ advice to patients that they could combine their prayers with their medications. Nogueira and colleagues (2023) suggest that nurses may assist persons living with HIV to cope with their condition by exploring such spiritual needs during care delivery.

The professional nurses provided self-management support to adults with TB-HIV coinfection by assessing and advising them on how to self-manage their symptoms, medications, nutrition and psychosocial problems, which is comparable to a study in Indonesia (Fauzi, Anggraini & Fatkhurohman 2021). Contrariwise, professional nurses did not provide nutrition-related self-management support to persons with chronic conditions in primary health settings in the United States of America (USA), UK and Canada (Beaudin et al. 2022). The findings may have differed because patients in resource-limited settings (like Ghana) experience more food insecurity problems than those in high-income countries such as the USA and the UK. Nonetheless, the professional nurses’ assessment appeared to be transversal to their self-management support for adults with TB-HIV coinfection because it formed the basis for identifying and addressing patients’ self-management problems (Rouleau et al. 2019). Dwarswaard and colleagues (2016) suggest that the dynamic and individual nature of self-management among persons with chronic conditions necessitates frequent assessment of their self-management support problems. The findings imply that professional nurses must hone their assessment skills to recognise, prioritise and provide the relevant self-management support that adults with TB-HIV coinfection require.

As found in a thematic synthesis of qualitative studies, the professional nurses enhanced the confidence of adults with TB-HIV coinfection in their ability to self-manage by educating and advising (counselling) them (Dwarswaard et al. 2016). Although patient education does not always lead to patients’ behaviour change, professional nurses’ education, encouragement, and advice appeared to have empowered the patients to deal with the impact of TB-HIV on their lives (Dwarswaard et al. 2016). Similarly, introducing persons living with chronic conditions to others with similar conditions can enhance their confidence to self-manage. However, professional nurses’ practice of pointing to the pile of medical records (folders) of other patients (without showing names or identifiers) could be an innovative approach to enhancing the confidence of adults with TB-HIV coinfection in settings with high levels of TB-HIV stigma.

Professional nurses’ collaboration with adults with TB-HIV coinfection, their relations and other healthcare providers was integral to their provision of self-management support. Comparable findings were reported in a scoping review of self-management support interventions provided by primary care nurses to persons living with chronic conditions (Beaudin et al. 2022). Evidently, the professional nurses’ collaboration with community health nurses, clinicians, dermatologists, nutritionists and psychologists ensured that the self-management problems of adults with TB-HIV coinfection could still be addressed even if they were beyond the capabilities and scope of practice of the professional nurses. Professional nurses must, therefore, consider such collaborative partnerships with relevant healthcare providers as a critical resource for providing self-management support to persons with chronic conditions such as adults with TB-HIV coinfection.

Professional nurses’ self-management support actions were feasible, equitable and acceptable to adults with TB-HIV coinfection and stakeholders. However, professional nurses’ improvisation to make TB-HIV coinfection self-management support possible is underreported in the literature. Such improvisations may pose a risk for unsafe variations in self-management support delivery and can be remedied by setting accepted self-management support procedures and providing resources for professional nurses to implement them (Abdulai et al. 2023).

Professional nurses’ self-management support was influenced by factors related to the patients, their relations, healthcare providers (professional nurses) and health facilities (TB-HIV clinic) (Duprez et al. 2018; Kelly et al. 2021). Contrary to the findings of this study, Duprez and colleagues (2018) found in Belgium that professional nurses’ availability of time was not associated with their self-management support behaviour. The variation in resource settings of both studies may have accounted for the difference. Nevertheless, the finding that experience, training and motivation facilitated professional nurses’ self-management support suggests that self-management support competencies could be engendered among professional nurses who lacked it.

### Strengths and limitation

This study provides insight into the experiences of professional nurses regarding the self-management problems of adults with TB-HIV coinfection and the self-management support that professional nurses provide them within the primary health context. The evidence regarding the feasibility, equity and acceptability of professional nurses’ self-management support can inform the production of evidence-based recommendations for self-management support. The limitation of this study is that it was conducted in three health facilities within the Greater Accra region of Ghana; hence, it may not capture the experiences of all professional nurses in all the regions of Ghana.

### Recommendations

Professional nurses improvised with limited resources to provide self-management support for adults with TB-HIV coinfection. Nurse managers and administrators of health facilities must support professional nurses in providing comprehensive self-management support to adults with TB-HIV coinfection by training, motivating, and equipping them with the relevant resources (including staff and funds).

## Conclusion

Professional nurses’ self-management support for adults with TB-HIV coinfection mainly entailed assessing the patients’ TB-HIV coinfection-related problems and counselling (advising) them on how to deal with their problems. The professional nurses supported the adults with TB-HIV coinfection to self-manage their symptoms, medications, nutrition and psychosocial problems. Nonetheless, patients’ low socioeconomic status, professional nurses’ lack of training and health facilities’ inadequate resources limit their self-management support. Professional nurses’ self-management support was feasible, equitable and acceptable, albeit with some improvisation and collaborative care with relations and other healthcare providers.
